# Supplementary material for: Structural basis for the inactivation of cytosolic DNA sensing by the vaccinia virus
Source: Nat Commun. 2022 Nov 18;13:7062. doi: 10.1038/s41467-022-34843-z (PMC9674614; doi:10.1038/s41467-022-34843-z)
Supplement: Supplementary file 1 — Supplementary Information [file 41467_2022_34843_MOESM1_ESM.pdf]

Supplementary Information for

Structural basis for the inactivation of cytosolic DNA sensing by the vaccinia virus

Angel Rivera-Calzada[1&], Raquel Arribas-Bosacoma[2,3&], Alba Ruiz-Ramos[1], Paloma Escudero-Bravo[1], Jasminka Boskovic[1], Rafael Fernandez-Leiro[1], Antony W. Oliver[2], Laurence H. Pearl[2,3*] and Oscar Llorca[1*]

1. Spanish National Cancer Research Centre (CNIO), Melchor Fernández Almagro 3, 28029 Madrid, Spain
2. Genome Damage and Stability Centre, School of Life Sciences, University of Sussex, Falmer, Brighton BN1 9RQ, UK
3. Division of Structural Biology, Institute of Cancer Research, Chester Beatty Laboratories, 237 Fulham Road, London SW1E 6BT, UK

[&] These authors contributed equally

* Correspondence to: LHP - laurence.pearl@sussex.ac.uk; OL - ollorca@cnio.es

Supplementary Figures

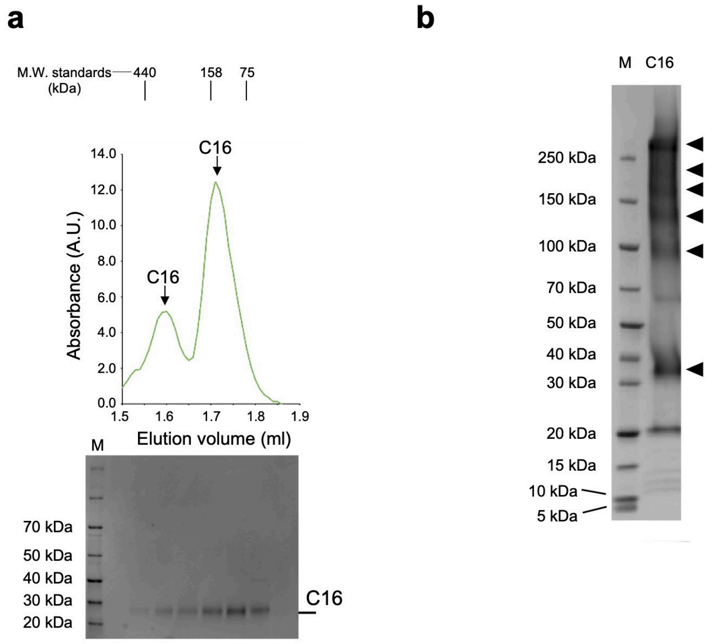

c

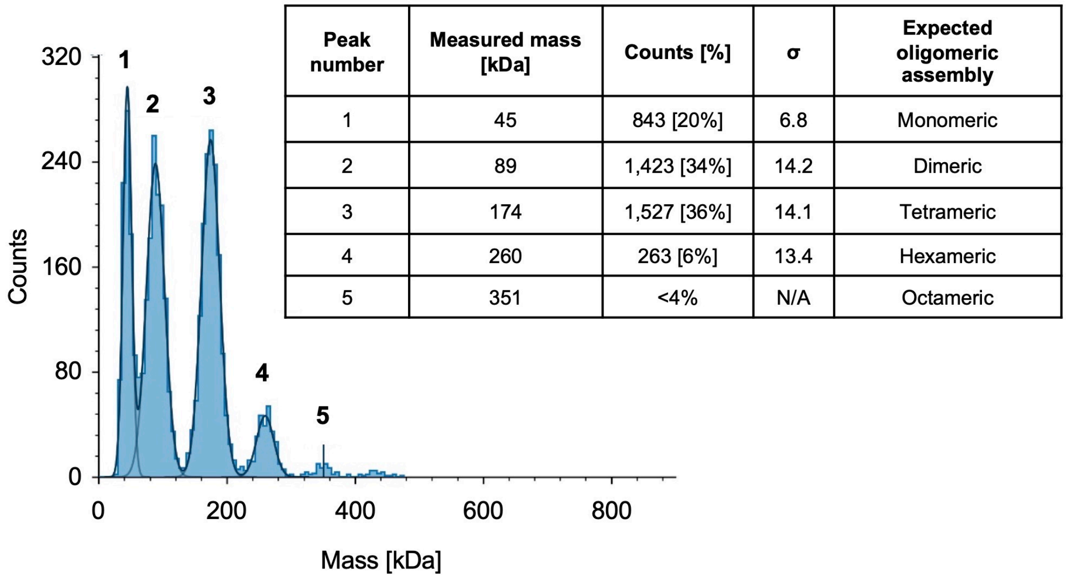

Peak number	Measured mass [kDa]	Counts [%]	σ	Expected oligomeric assembly
1	45	843 [20%]	6.8	Monomeric
2	89	1,423 [34%]	14.2	Dimeric
3	174	1,527 [36%]	14.1	Tetrameric
4	260	263 [6%]	13.4	Hexameric
5	351	<4%	N/A	Octameric

Supplementary Fig. 1. Purification and oligomeric state of C16

a SEC of purified C16. Selected fractions were analysed by SDS-PAGE (bottom panel). The elution volume for the peaks of standard proteins Ferritin (440 kDa), Aldolase (158 kDa) and Conalbumin (75 kDa) are indicated at the top of the chromatogram (M.W. stands for Molecular

Weight). SEC of C16 was repeated at least five times with similar results. Molecular weight marker (M) are shown.

b Purified C16 protein exhibits a tendency to oligomerize when analysed by SDS-PAGE under less-stringent denaturing conditions by avoiding boiling the mix of protein and loading buffer. Lanes displayed are molecular weight marker (M) and purified C16. Triangles indicate the different oligomers formed by C16. This was repeated at least three times with similar results.

c Mass distribution histogram obtained using mass photometry for the purified C16 protein. Details for each of the peaks are shown in the accompanying table. Purified C16 forms different oligomeric assemblies as observed during its purification and by cryoEM. σ corresponds to the standard deviation of the fit for each peak.

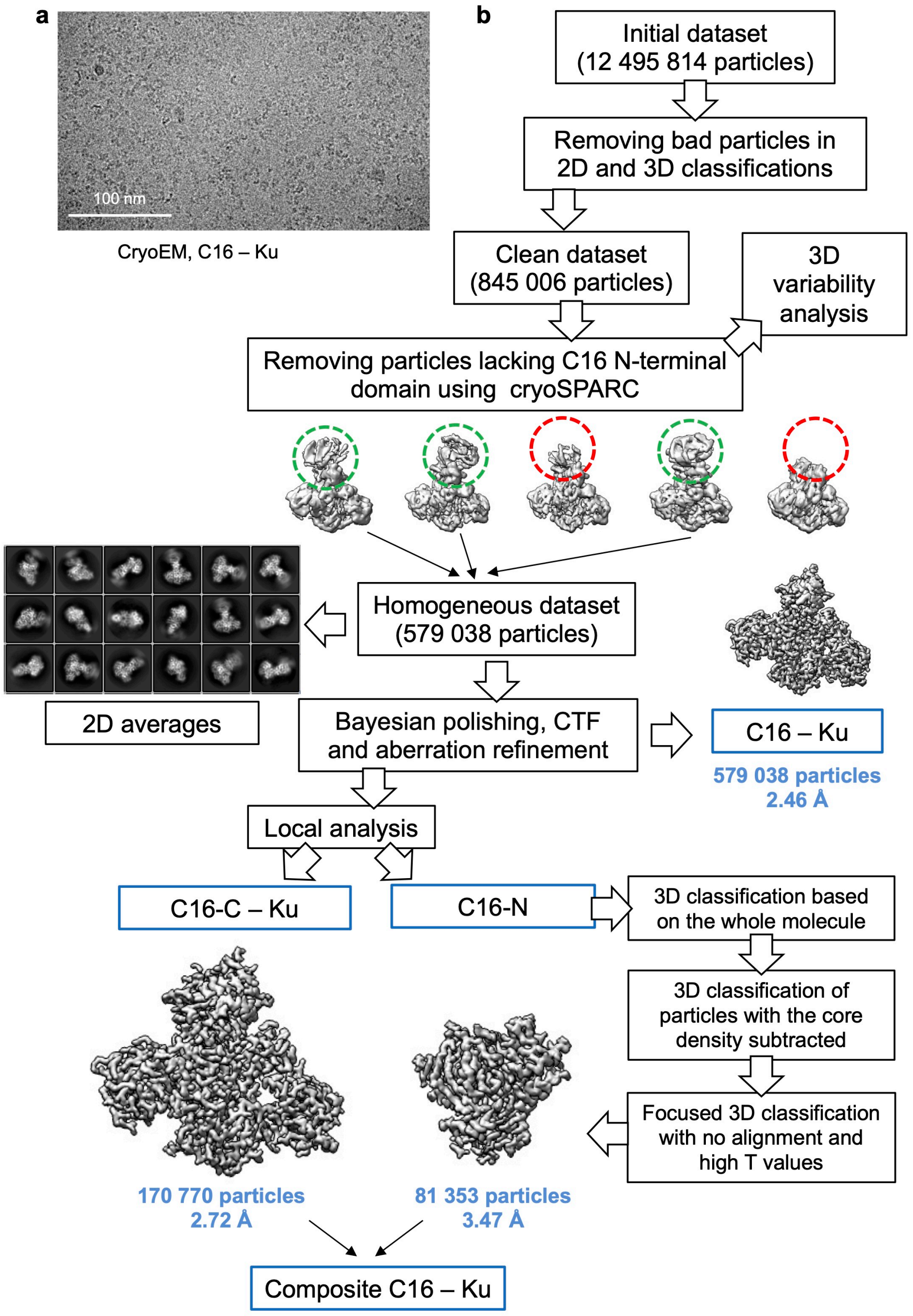
a
100 nm
CryoEM, C16 – Ku
b
Initial dataset
(12 495 814 particles)
Removing bad particles in
2D and 3D classifications
Clean dataset
(845 006 particles)
3D
variability
analysis
Removing particles lacking C16 N-terminal
domain using cryoSPARC
Homogeneous dataset
(579 038 particles)
2D averages
Bayesian polishing, CTF
and aberration refinement
C16 – Ku
579 038 particles
2.46 Å
Local analysis
C16-C – Ku
C16-N
3D classification based
on the whole molecule
3D classification of
particles with the core
density subtracted
Focused 3D classification
with no alignment and
high T values
170 770 particles
2.72 Å
81 353 particles
3.47 Å
Composite C16 – Ku

c

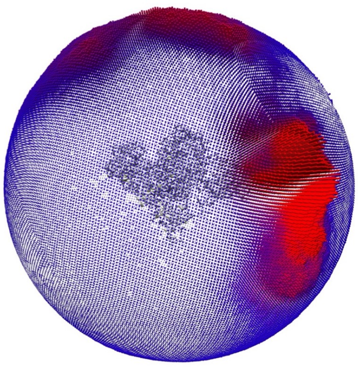

d

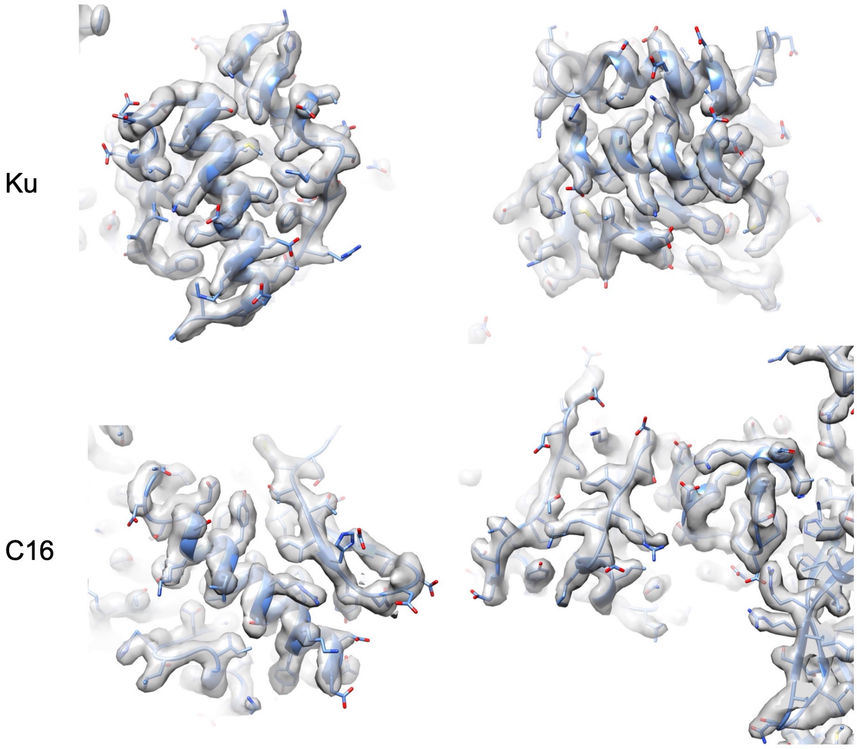

Supplementary Fig. 2. CryoEM and image processing workflow for the C16 – Ku complex

a A representative cryoEM field of a total of 13,216 movies collected (see Supplementary Table 1 for a complete list of cryoEM data collection parameters), showing individual particles of the C16 – Ku complex.

b Image processing workflow of the cryoEM data for the C16 – Ku complex. Image processing and classification strategies for the cryo-EM data of the C16 – Ku complex. Tools available in Relion 4.0[1,2] and cryoSPARC[3] were used to obtain homogeneous groups of particles that could be refined to high-resolution. cryoSPARC was used to reveal classes of particles in which the N-terminal domain of C16 was absent or poorly defined (encircled in red). The region corresponding to C16 C-terminal domains bound to Ku could be refined to high-resolution (C16 – Ku and C16-

C – Ku maps). The C16-C – Ku map, albeit having a worse overall resolution value than the C16 – Ku map, showed improved detail at two regions: the bridge that encircles the DNA binding region in Ku, and the C-terminal domain of the C16 molecule that protrudes in the DNA binding region. N-terminal domains in C16 are flexibly attached to the rest of the molecule. Therefore, we designed a strategy that combined 3D classifications using masks of the whole molecule, but also 3D classifications using density-subtracted images that only contain density corresponding to C16-N. Finally focused 3D classification combined with high T values revealed a subset that was refined to 3.5 Å resolution (C16-N map). The C16-C – Ku and C16-N maps were merged into a single composite C16 – Ku map. Representative 2D averages are shown.

c Angular distribution of particles used in the final reconstruction for the C16-C – Ku complex.

d Representative regions of the cryoEM map of the C16-C – Ku complex shown as a transparency, with the structural model, in blue colour, fitted. Top panels show regions of the Ku molecule in the complex. Bottom panels show regions of the C16 molecule in the complex.

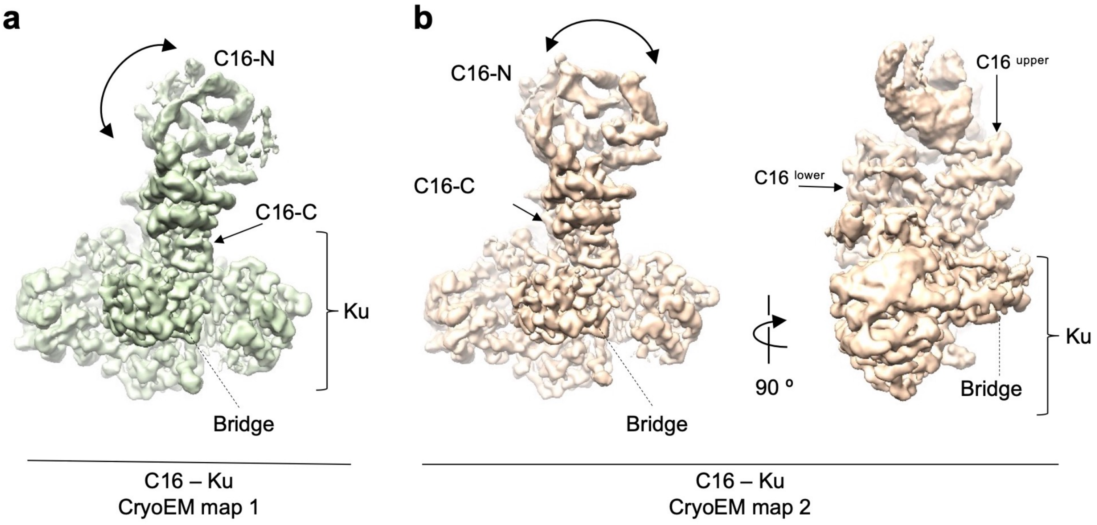

Supplementary Fig. 3. Flexibility of the C16 – Ku complex

a, b Two selected cryoEM volumes of the C16 – Ku complex, in green and light orange colour, highlighting the flexibility of the C16 N-terminal region. For one of the volumes, two views are shown and the two C16 monomers labelled (right panel).

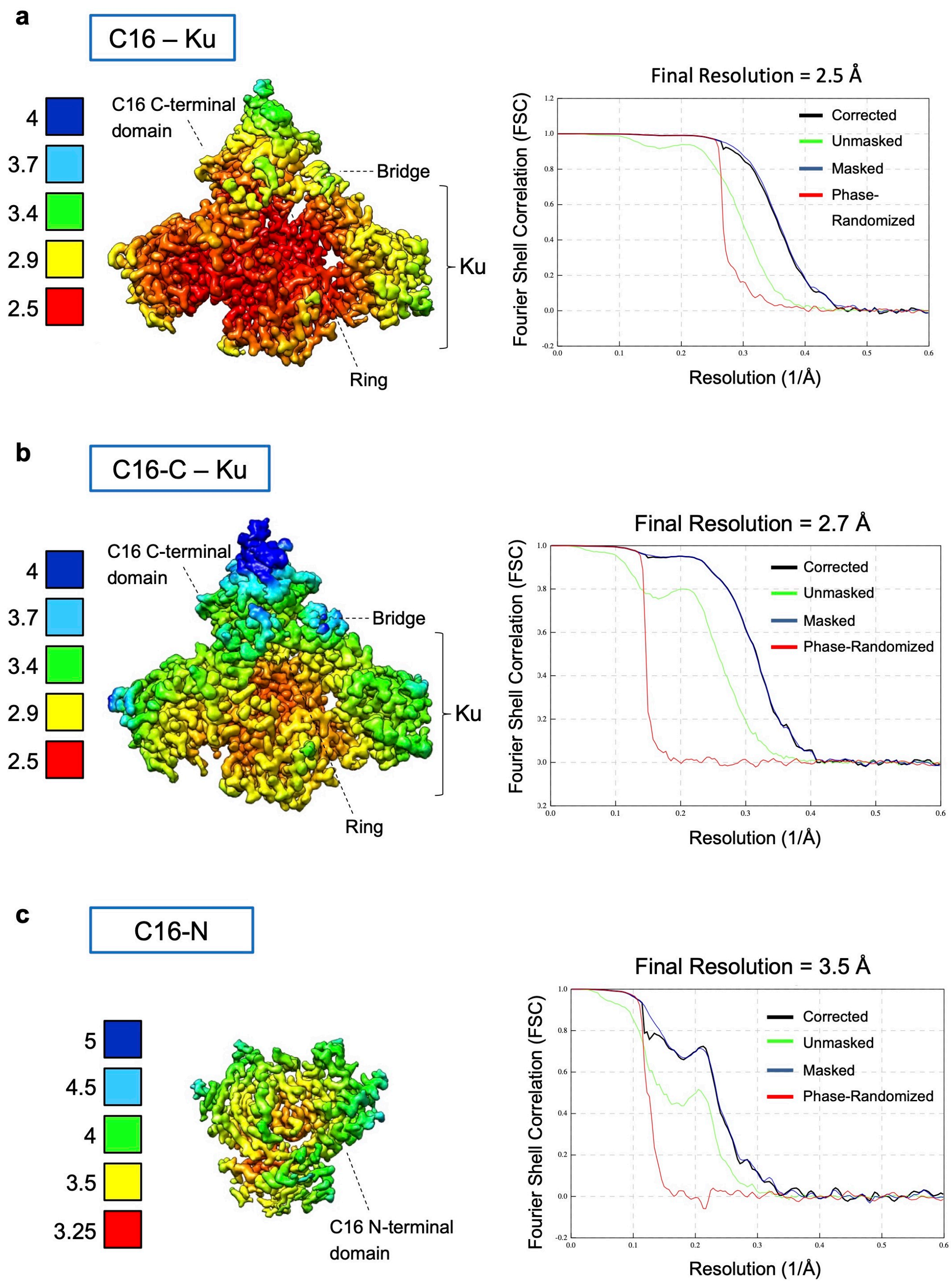

Supplementary Fig. 4. Resolution estimates of the cryoEM maps

a Right panel - FSC curves for the estimation of the average resolution of the cryoEM map C16 – Ku. Left panel - Local resolution map estimate for the cryoEM map of C16 – Ku.

b As in a but for the cryoEM map C16-C – Ku.

c As in a but for the cryoEM map C16-N.

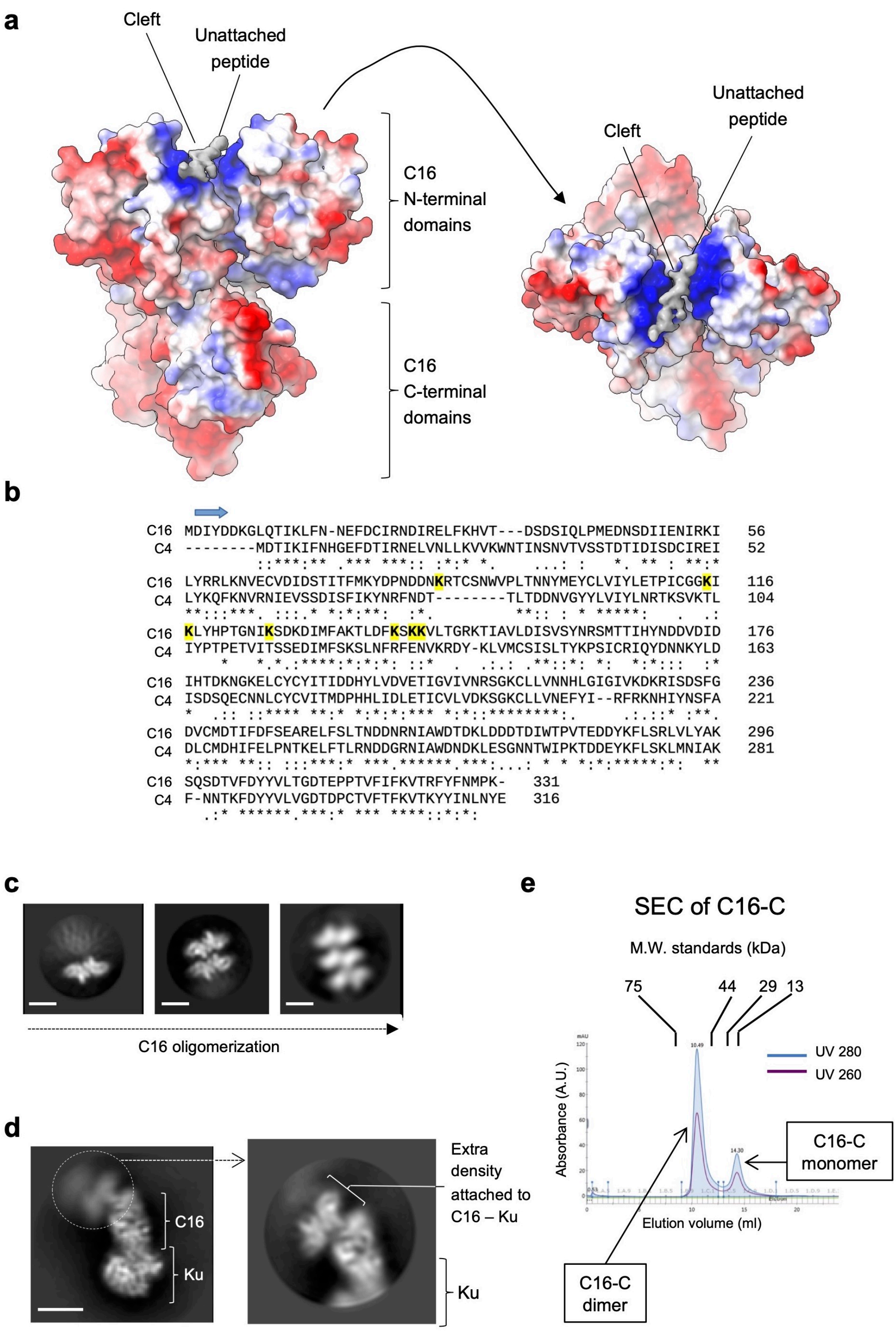

Supplementary Fig. 5. C16 structure and oligomerization

a Coulombic electrostatic potential of the C16 homodimer. The N-terminal domains form a basic cleft where we identified an unattached density not accounted by the C16 molecules forming the

dimer. This density could come from an adjacent C16 dimer as observed in 2D classes corresponding to the C16 – Ku complex that contain additional C16 molecules bound to it.

b Sequence alignment of C16 and C4. Both sequences exhibit a high-degree of identity. In C16 residues at the N-terminus form a β-strand that is absent in C4, and it is indicated with a blue arrow. Lysines located at the basic cleft (see panel a) are highlighted in bold and yellow. Alignment was performed using Clustal Omega (1.2.4)(https://www.ebi.ac.uk/Tools/msa/clustalo/)[4].

c 2D averages of several oligomeric species for C16. Scale bar corresponds to 5 nm.

d 2D averages of C16 – Ku complexes where C16 appears to oligomerize as part of a complex between Ku and C16. Scale bar corresponds to 5 nm.

e C16-C can dimerise in the absence of the N-terminal domain. Superdex 75 10/300 GL SEC profile for cterm-C16, which comprises residues from 157-331 and has an expected molecular weight of 23 kDa for a monomer and 46 kDa for dimer. The elution volumes corresponding to standard proteins Conalbumin (75 kDa), Ovalbumin (44 kDa), Carbonic Anhydrase (29 kDa) and Ribonuclease A (13.7 kDa) are indicated on top of the chromatogram.

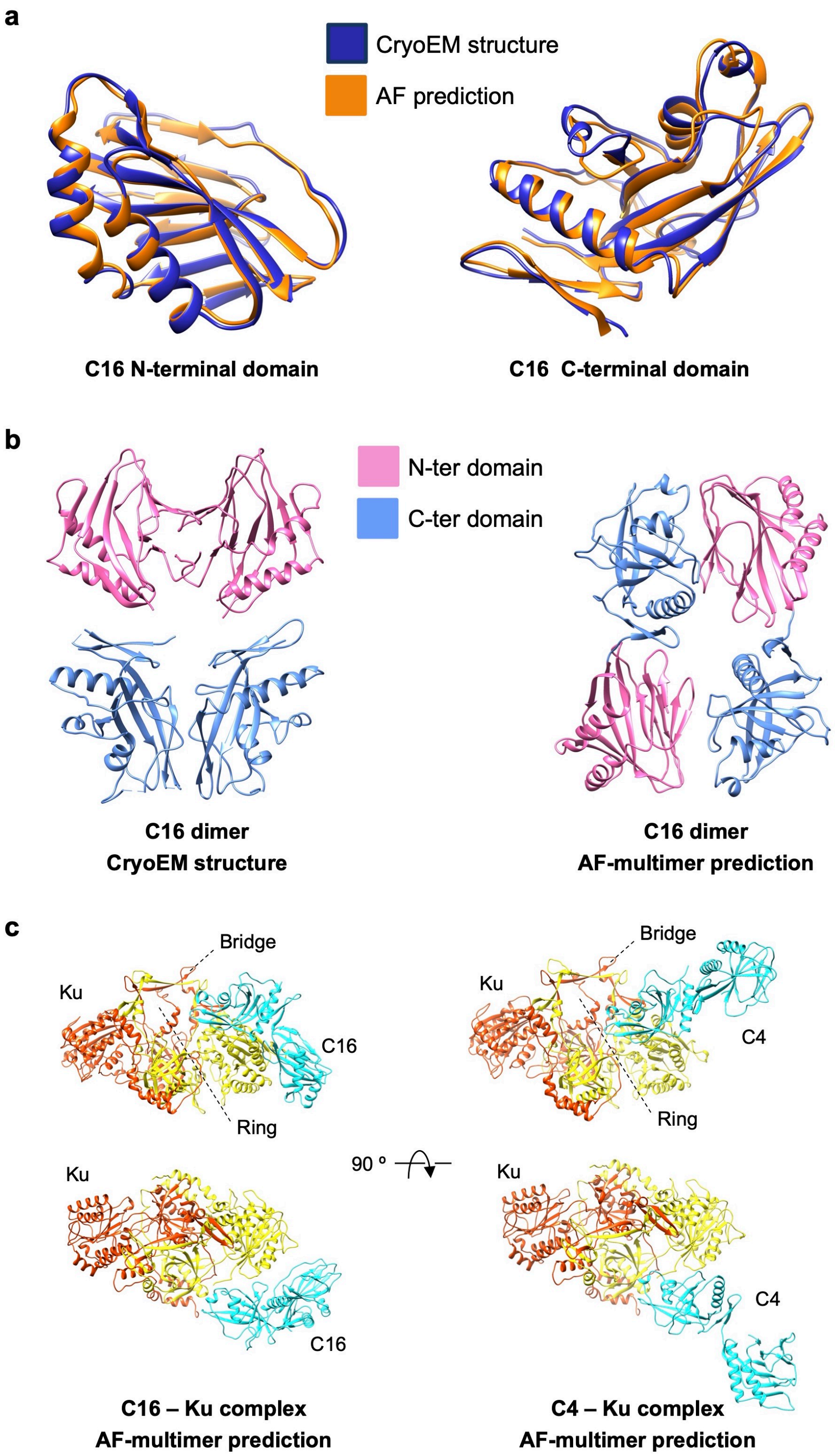

Supplementary Fig. 6. Comparison between AF prediction and experimental structures

a Comparison between the cryoEM structure of C16 N-terminal and C-terminal domains (blue colour) and a representative top-ranked AlphaFold (AF) prediction[5] (orange colour). Each domain is analysed separately because they are flexibly connected.

b Comparison between the cryoEM structure and AF-multimer (https://www.deepmind.com/publications/protein-complex-prediction-with-alphafold-multimer)[6] predictions of C16 dimers. For the cryoEM structure, N- and C-terminal domains (coloured in pink and light blue respectively) are showed separately because we find several relative orientations and also to highlight the dimerization interface for each domain. All top-ranked AF predictions suggest the dimerization between the N-terminal and C-terminal domain, and the figure shows a representative top-ranked AF.

c AF-multimer[6] predictions of C16 – Ku and C4 – Ku complexes. Predictions using 2 copies of C4 and C16 to allow prediction of dimers did not generate any prediction where C4 or C16 contacted Ku. When predictions were launched using only 1 chain of C4 and C16, some of the top-ranked solutions positioned the proteins in the vicinity of Ku, but none reproduced the interactions determined experimentally. Figure shows a representative top-ranked prediction for each protein.

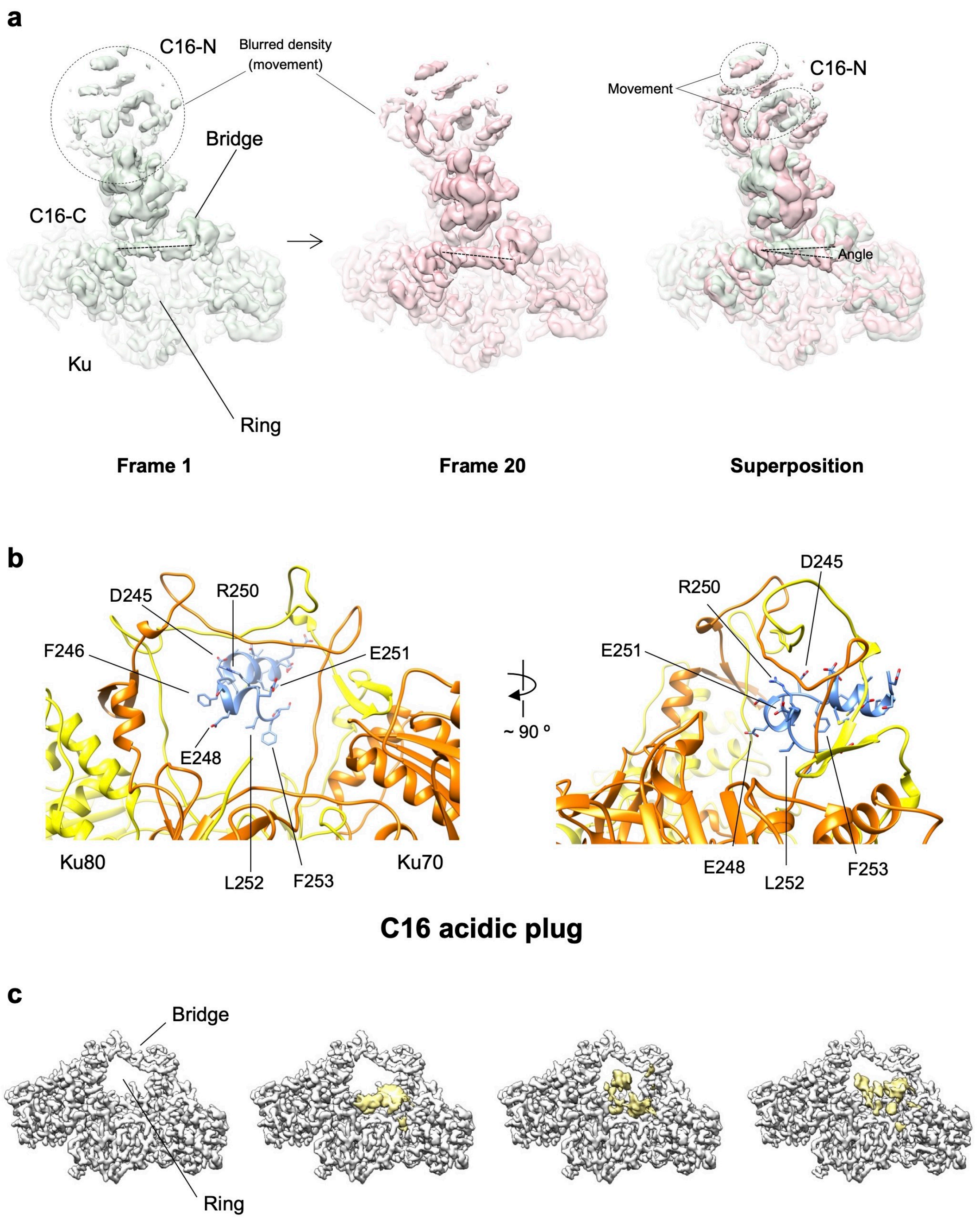

Supplementary Fig. 7. Structure and heterogeneity in the Ku ring where C16 binds

a The interaction of C16-C with Ku results in some deformation of the bridge forming the DNA binding ring, as revealed by 3D variability analysis performed in cryoSPARC[3] (Supplementary Movies 1, 2 and 3). This figure highlights the conformational changes in the Ku bridge by showing the first and last frame of one of these movies. For the Ku bridge we draw a line along

the density for the bridge to highlight the change in position. Density for C16-N is blurred due to its flexibility and it does not occupy the same position in each of the frames represented.

b Two views showing details of the C16 acidic ‘plug’ inserting in the Ku ring. Some residues are indicated.

c Particles from the C16 – Ku map were also analysed using focused 3D classification with local masks, not allowing movements during classification, and using a high T value of 200 in Relion 4.0[1]. This analysis revealed different degrees of filling within the ring region in Ku that is at the opposite end to where C16 binds, with a density that exhibited a random orientation. The density was tentatively assigned to the N-terminus of Ku70 because it showed continuity with the rest of the chain in Ku70. This N-terminal region is enriched in negatively charged amino acids which could interact with the positively charged regions at the interior of the DNA binding region in Ku. Figures show several of the maps after3D classification, with the variable density coloured in yellow.

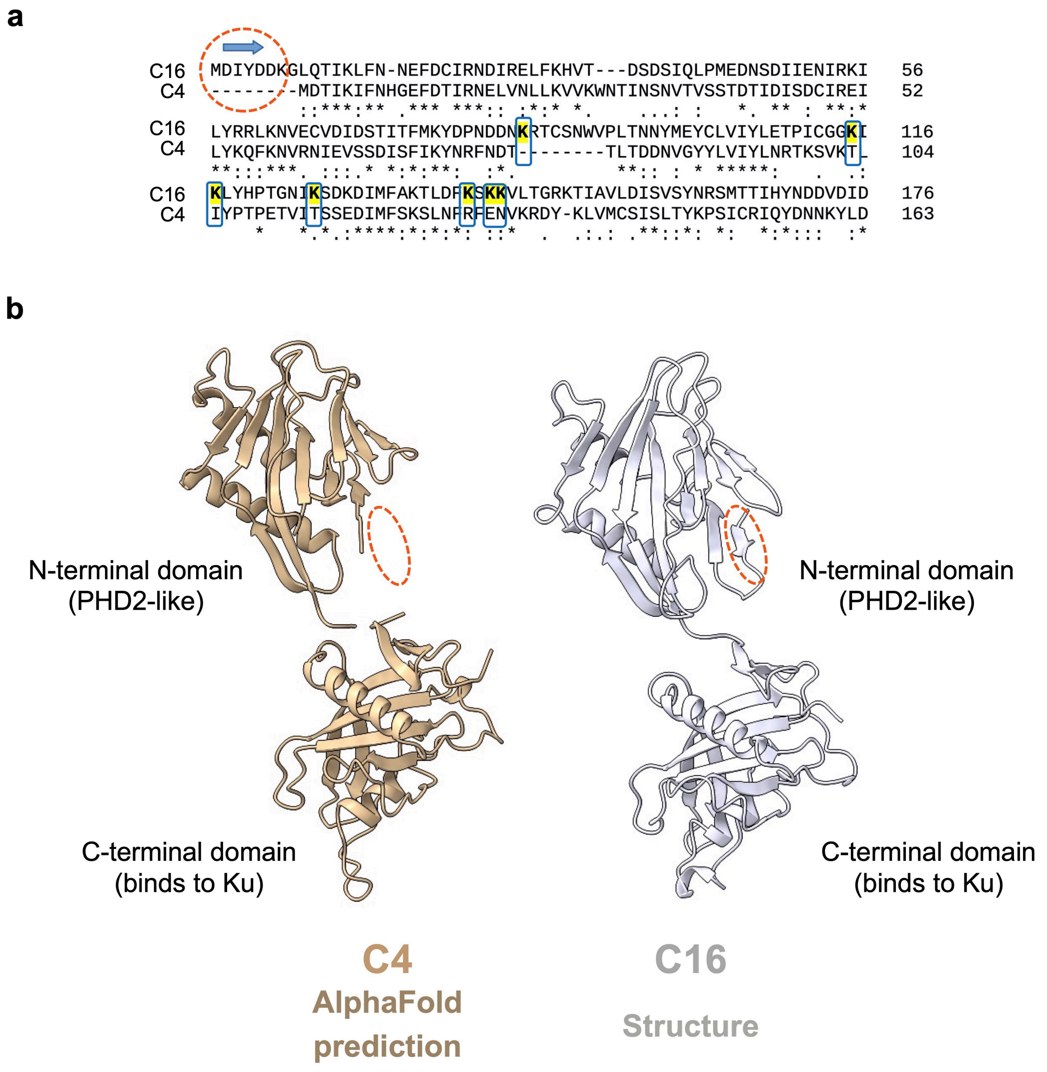

Supplementary Fig. 8. Comparison between the structures of C4 and C16

a Sequence alignment of the N-terminal region of C16 and C4. A β-strand present in C16 and absent in C4 is indicated with a blue arrow and within a red dashed circle. Several lysine residues at the N-terminus of C16 form the basic cleft (see panel Supplementary Fig. 5) but are absent in C4 and they are highlighted in bold and yellow within a blue square. Alignment was performed using Clustal Omega (1.2.4)(https://www.ebi.ac.uk/Tools/msa/clustalo/)[4].

b Comparison between the structure of C16 and the predicted structure of C4 using AlphaFold[5]. An N-terminal β-strand is present in C16 but absent in C4 and its presence and absent is highlighted with a dashed circle.

a

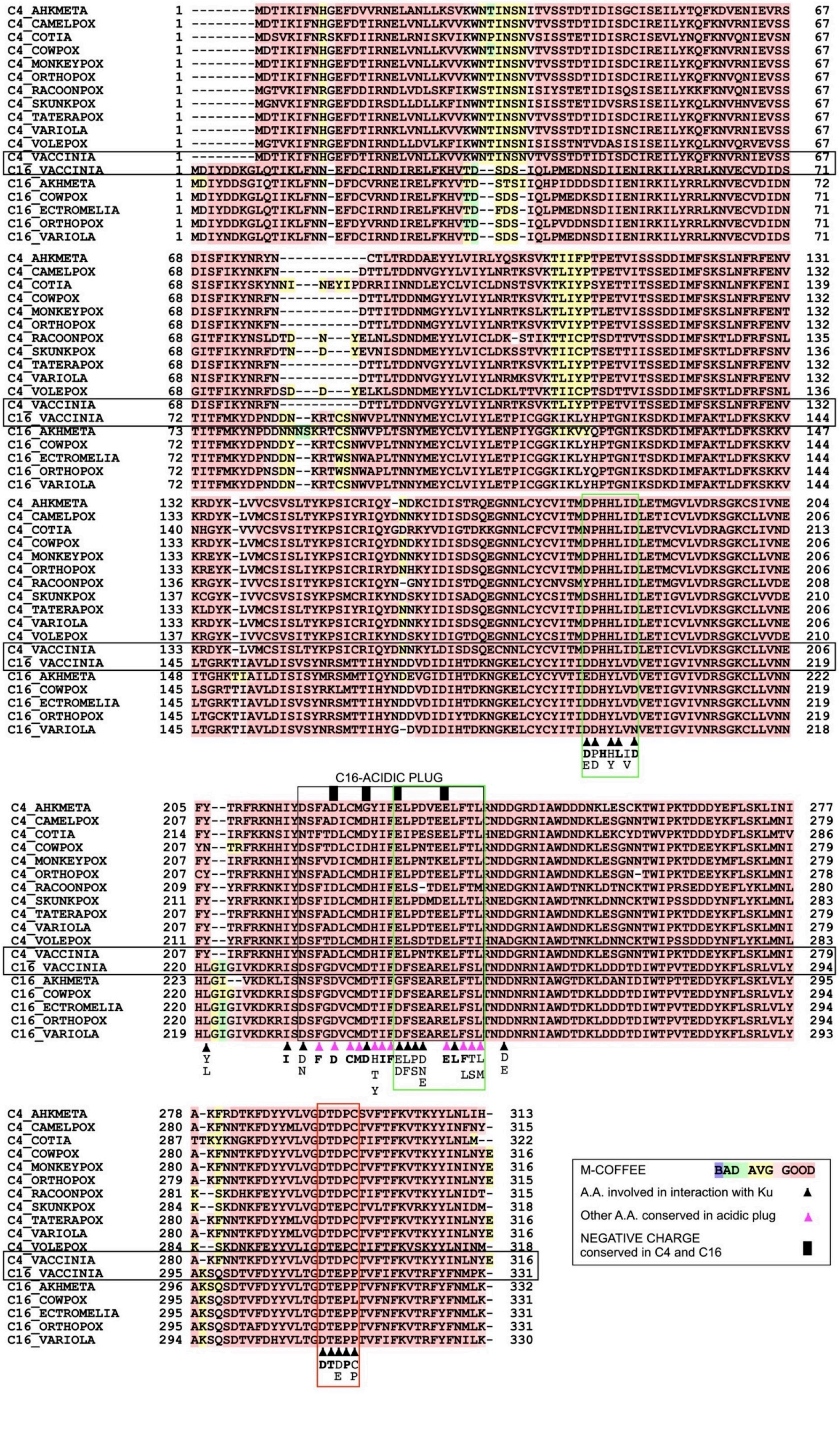
C16-ACIDIC PLUG
DPHHLID
ED Y V
DTDPC
E P
M-COFFEE
BAD AVG GOOD
A.A. involved in interaction with Ku
Other A.A. conserved in acidic plug
NEGATIVE CHARGE
conserved in C4 and C16

b

	NCBI REF. SEQ	UNIPROT ACCESSION	UNIPROT CODE	GENE NAME	VIRUS
C4_Akmeta	YP_010085477.1	-	-	IL-1 receptor antagonist	Akhmeta
C4_Camelpox	NP_570412.1	Q8V2Z5	Q8V2Z5_CAMPM	CamMLVgp022	Camelpox
C4_Cotia	YP_005296213.1	H6TAH8	H6TAH8_9POXV	IL-1 receptor antagonist	Cotia (SPAn232)
C4_Cowpox	NP_619822.1	Q8QN29	Q8QN29_CWPXB	CPXV033	Cowpox
C4_Monkeypox	YP_010377015.1	-	-	C4L/C10L-like family protein	Monkeypox
C4_Orthopox	YP_010085694.1	-	-	Putative C4L protein	Orthopox
C4_Racoonpox	YP_009143333.1	A0A0G3FZW3	A0A0G3FZW3_RACVI	IL-1 receptor antagonist	Racoonpox
C4_Skunkpox	YP_009282717.1	A0A1C9KBH6	A0A1C9KBH6_9POXV	IL-1 receptor antagonist	Skunkpox
C4_Taterapox	YP_717332.1	Q0NPI6	Q0NPI6_9POXV	TATV_DAH68_025	Taterapox
C4_Variola	NP_042055.1	P34012	C4_VAR67	VARVgp011	Variola (Smallpox)
C4_Volepox	YP_009281771.1	A0A1C9KC28	A0A1C9KC28_9POXV	IL-1 receptor antagonist	Volepox
C4_Vaccinia	YP_232906.1	P17370	C4_VACCW	VACWR024	Vaccinia
C16_Vaccinia	YP_232892.1	H2DUG7	H2DUG7_9POXV	VACWR010	Vaccinia
C16_Akmeta	YP_010085466.1	-	-	IL-1 receptor antagonist	Akhmeta
C16_Cowpox	NP_619811.1	Q8QN39	Q8QN39_CWPXB	CPXV022	Cowpox
C16_Ectromelia	NP_671529.1	Q8JLI8	Q8JLI8_9POXV	ECTVgp011	Ectromelia
C16_Orthopox	YP_010085683.1	-	-	C10L protein	Orthopox
C16_Variola	NP_042047.1	P33861	C10_VAR67	VARVgp003	Variola (Smallpox)

Supplementary Fig. 9. Multiple amino acid sequence alignment of C4 and C16 proteins from the poxviridae family

a Output from the M-COFFEE server[7], hand-annotated to incorporate information about C16 dimer - Ku70/80 interactions obtained using the online tool PDBsum (http://www.ebi.ac.uk/thornton-srv/databases/cgi-bin/pdbsum/GetPage.pl?pdbcode=index.html). The alignment is coloured according to the internal algorithm of M-COFFEE, where regions coloured yellow/orange/red indicate that the residues are likely to be correctly aligned (see associated key for additional details). Amino acids involved in interactions with Ku are marked with filled triangles and those conserved in the acidic plug but not directly involved in the interaction with Ku are marked with a pink triangle. The predominant amino acid found at each site of interaction is also shown, with those shown in bold text indicating absolute/strong conservation of identity across both C4 and C16 proteins. The residue range corresponding to the described ‘acidic plug’ of C16 is enclosed by a black rectangle, with regions of conserved negative charge indicated by blocks coloured black (strong conservation). Residues from the acid plug that are directly inside de Ku ring or in contact with Ku80 are highlighted within a green box. Other regions of interaction with Ku outside the acidic plug are indicated with green and red boxes. Black boxes serve to highlight the amino acid sequences of the C4 and C16 proteins from Vaccinia described in this study.

b Table providing accession codes and additional information about each amino acid sequence used to generate the multiple sequence alignment.

Supplementary Tables

Supplementary Table 1. CryoEM data collection parameters

CryoEM data collection parameters		
Data set name	CNIO, Spain	Leicester, UK
Microscope	JEM-2200FS (JEOL)	Titan Krios G3 (Thermo Fisher Scientific)
Camera	K3 in counting mode (Gatan)	K3 in counting mode (Gatan)
Magnification	40K	105K
Stage Tilt	0°	0°
Voltage (kV)	200	300
Number of Movies	827	13,216
Exposure time (s)	4	2
Number of frames	40	50
Dose per frame (e^-/Å^2/frame)	1	1
Total Exposure (e^-/Å^2)	40	50
Defocus range (μm)	-1.3 to -2.3	-0.6 to -2.4
Original pixel size (Å/pixel)	0.9773	0.835

Supplementary Table 2. CryoEM image processing parameters

CryoEM image processing parameters				
Data set name	CNIO, Spain	Leicester, UK		
Macromolecular Complex	C16 – Ku	C16 – Ku	C16-C – Ku	C16-N
Processing Pixel Size (Å/pixel)	0.9773	0.835	0.835	0.835
Box Size (pixels)	240 x 240	304 x 304	304 x 304	304 x 304
Imposed Symmetry	C1	C1	C1	C1
Number of Particles	242,817	579,038	170,770	81,353
FSC Threshold	0.143	0.143	0.143	0.143
Resolution (Å)	4.04	2.46	2.72	3.47
Resolution Range (Å)	N/A	2.5-4	2.5-4	3.25-5
Map Sharpening B-factor ($Å^2$)	N/A	-65	-65	-88

Supplementary Table 3. Model building and refinement parameters

Model building and refinement			
**Deposited CryoEM model**<br>EMDB code	C16-C – Ku<br>EMD-15415	C16-N<br>EMD-15414	Composite map (C16 – Ku)<br>EMD-15416
**Initial model**	AlphaFold2 for C16<br>PDB 1JEY for Ku	AlphaFold2	AlphaFold2 for C16<br>PDB 1JEY for Ku
**Model composition**			
Chains	4	2	4
Non-hydrogen atoms	11,157	2,632	13,762
Protein residues	1,384	322	1,703
Ligands	0	0	0
**R.m.s. deviations**			
Bond lengths (Å)	0.003	0.002	0.003
Bond angles (°)	0.460	0.600	0.486
**Validation**			
MolProbity score	1.48	1.91	1.62
Clash score	4.35	8.85	6.46
Poor rotamers (%)	0.00	0.00	0.26
**Ramachandran plot**			
Favored (%)	96.14	93.4	96.10
Allowed (%)	3.79	6.60	3.90
Disallowed (%)	0.07	0.00	0.00
**Deposited PDB code**	8AG4	8AG3	8AG5

Supplementary Table 4. Sequence of DNA fragments used in this work

**Fragment (5'→3')**	**Sequence**
Twin-StrepTag gBlocks™ gene fragment (177 bp)	GTTTAACTTTAAGAAGGAGATATACC**ATG**GGCGGAGAGAATCTTTATTTTCAGGGCtggagccacccgcagttcgaaaagggaggaggatctggaggaggatcaggtggttcaagcgcgtggagccacccgcagttcgaaaag**TGATAA**TTAACCTAGGCTGCTGCCACCGCTGAG
C16_FL_RF_Fwd (53 nt)	TAACTTTAAGAAGGAGATATACC**ATG**GATATTTACGACGATAAAGGTCTACAG
C16_FL_RF_Rev (56 nt)	ccaGCCCTGAAAATAAAGATTCTCTCCGCCTTTCGGCATATTAAAGTAAAATCTAG

Note: Underlined nucleotides correspond to sequences present in the original pET-52b(+) plasmid. In bold the ATG start codon. In orange sequence of the TEV cleavage site. In lower-case sequence of the Twin-Strep tag. In bold red stop codons. In blue sequences of the C16 protein.

Supplementary references

1. Kimanius, D., Dong, L., Sharov, G., Nakane, T. & Scheres, S.H.W. New tools for automated cryo-EM single-particle analysis in RELION-4.0. *Biochem J* **478**, 4169-4185 (2021).

2. Zivanov, J. et al. New tools for automated high-resolution cryo-EM structure determination in RELION-3. *Elife* **7**(2018).

3. Punjani, A., Rubinstein, J.L., Fleet, D.J. & Brubaker, M.A. cryoSPARC: algorithms for rapid unsupervised cryo-EM structure determination. *Nat Methods* **14**, 290-296 (2017).

4. Sievers, F. et al. Fast, scalable generation of high-quality protein multiple sequence alignments using Clustal Omega. *Mol Syst Biol* **7**, 539 (2011).

5. Jumper, J. et al. Highly accurate protein structure prediction with AlphaFold. *Nature* **596**, 583-589 (2021).

6. Evans, R. et al. Protein complex prediction with AlphaFold-Multimer. *bioRxiv*, 2021.10.04.463034 (2022).

7. Notredame, C., Higgins, D.G. & Heringa, J. T-Coffee: A novel method for fast and accurate multiple sequence alignment. *J Mol Biol* **302**, 205-17 (2000).
