# Supplementary material for: Structural basis for the inactivation of cytosolic DNA sensing by the vaccinia virus
Source: Nat Commun. 2022 Nov 18;13:7062. doi: 10.1038/s41467-022-34843-z (PMC9674614; doi:10.1038/s41467-022-34843-z)
Supplement: Supplementary file 3 — Description of Additional Supplementary Files [file 41467_2022_34843_MOESM3_ESM.pdf]

Description of Additional Supplementary Files

File Name: Supplementary Movie 1
Description: **Side view of the C16 – Ku and variability analysis.** Movie corresponds to one view of the main component resulting from the 3D variability analysis performed in cryoSPARC. This analysis was performed in the subset of 579,000 particles before further classification in order to improve details and resolution of the C16-C – Ku complex.

The movie shows the C16 – Ku complex from its side to highlight the region of the ring and the bridge of Ku. The movie reveals the high degree of flexibility present in the C16 – Ku complex. This flexibility is mostly due to the swinging movement of C16 when bound to Ku, and the movie shows deformations in the bridge of the Ku heterodimer. In addition, the C16 N-terminal and C-terminal domains are flexibly connected, and this also contributes to the whole flexibility of the complex.

File Name: Supplementary Movie 2
Description: **Tilted view of the C16 – Ku and variability analysis.** Movie corresponds to one view of the main component resulting from the 3D variability analysis as in Supplementary Movies 1 and 3, but showing a tilted view of the complex where the flexibility of C16-N is better appreciated.

File Name: Supplementary Movie 3
Description: **Top view of the C16 – Ku and variability analysis.** Movie corresponds to one view of the main component resulting from the 3D variability analysis as in Supplementary Movies 1 and 2, but showing a top view of the complex where the flexibility of the bridge, and the flexibility in the attachment of C16 to Ku is better appreciated.
