# Supplementary material for: Structural basis for the inactivation of cytosolic DNA sensing by the vaccinia virus
Source: Nat Commun. 2022 Nov 18;13:7062. doi: 10.1038/s41467-022-34843-z (PMC9674614; doi:10.1038/s41467-022-34843-z)
Supplement: Supplementary file 7 — Reporting Summary [file 41467_2022_34843_MOESM7_ESM.pdf]

nature portfolio

Corresponding author(s): Laurence H Pearl, Oscar Llorca

Last updated by author(s): Oct 3, 2022

Reporting Summary

Nature Portfolio wishes to improve the reproducibility of the work that we publish. This form provides structure for consistency and transparency in reporting. For further information on Nature Portfolio policies, see our Editorial Policies and the Editorial Policy Checklist.

Statistics

For all statistical analyses, confirm that the following items are present in the figure legend, table legend, main text, or Methods section.

n/a	Confirmed	
☐	☒	The exact sample size (*n*) for each experimental group/condition, given as a discrete number and unit of measurement
☐	☒	A statement on whether measurements were taken from distinct samples or whether the same sample was measured repeatedly
☒	☐	The statistical test(s) used AND whether they are one- or two-sided<br>*Only common tests should be described solely by name; describe more complex techniques in the Methods section.*
☒	☐	A description of all covariates tested
☒	☐	A description of any assumptions or corrections, such as tests of normality and adjustment for multiple comparisons
☒	☐	A full description of the statistical parameters including central tendency (e.g. means) or other basic estimates (e.g. regression coefficient) AND variation (e.g. standard deviation) or associated estimates of uncertainty (e.g. confidence intervals)
☒	☐	For null hypothesis testing, the test statistic (e.g. *F*, *t*, *r*) with confidence intervals, effect sizes, degrees of freedom and *P* value noted<br>*Give P values as exact values whenever suitable.*
☒	☐	For Bayesian analysis, information on the choice of priors and Markov chain Monte Carlo settings
☒	☐	For hierarchical and complex designs, identification of the appropriate level for tests and full reporting of outcomes
☒	☐	Estimates of effect sizes (e.g. Cohen's *d*, Pearson's *r*), indicating how they were calculated

Our web collection on statistics for biologists contains articles on many of the points above.

Software and code

Policy information about availability of computer code

Data collection	Data collection for the Leicester data set was performed using the EPU software (ThermoFisher Scientific). Data collection for the CNIO data set was performed using the SerialEM software (https://bio3d.colorado.edu/SerialEM/). This information is indicated in Methods
Data analysis	For Data analysis the following standard software packages were used: RELION, CTFFIND4, cryoSPARC, DeepEMhancer, Phenix, COOT and UCSF Chimera. All these softwares are cited in the manuscript.

For manuscripts utilizing custom algorithms or software that are central to the research but not yet described in published literature, software must be made available to editors and reviewers. We strongly encourage code deposition in a community repository (e.g. GitHub). See the Nature Portfolio guidelines for submitting code & software for further information.

Data

Policy information about availability of data

All manuscripts must include a data availability statement. This statement should provide the following information, where applicable:

- Accession codes, unique identifiers, or web links for publicly available datasets
- A description of any restrictions on data availability
- For clinical datasets or third party data, please ensure that the statement adheres to our policy

The cryoEM maps for the C16 – Ku complex generated in this study have been deposited in the Electron Microscopy Data Base (EMDB) under accession codes

EMD-15414, EMD-15415 and EMD-15416 (https://www.ebi.ac.uk/emdb/). The atomic models for the C16 – Ku complex generated in this study have been deposited in the Protein Data Base (PDB) under accession codes 8AG3, 8AG4 and 8AG5 (https://www.rcsb.org/).

Human research participants

Policy information about studies involving human research participants and Sex and Gender in Research.

Reporting on sex and gender	n/a
Population characteristics	n/a
Recruitment	n/a
Ethics oversight	n/a

Note that full information on the approval of the study protocol must also be provided in the manuscript.

Field-specific reporting

Please select the one below that is the best fit for your research. If you are not sure, read the appropriate sections before making your selection.

☒ Life sciences ☐ Behavioural & social sciences ☐ Ecological, evolutionary & environmental sciences

For a reference copy of the document with all sections, see nature.com/documents/nr-reporting-summary-flat.pdf

Life sciences study design

All studies must disclose on these points even when the disclosure is negative.

Sample size	12 495 814 particles of the C16 - Ku70/Ku80 complex were extracted from 13 216 cryoEM movies collected. Details can be found in Supplementary Figure 2b and Supplementary Tables 1 and 2.
Data exclusions	After removing bad particles and classifying to find the most homogeneous subsets, 3 subsets of 579,038 particles, 170,770 particles, and 81,353 particles respectivley were used for 3D image processing. Details can be found in Supplementary Figure 2b and Supplementary Table 2.
Replication	This does not apply to the cryoEM experiments described in this work
Randomization	This does not apply to the cryoEM experiments described in this work
Blinding	This does not apply to the cryoEM experiments described in this work

Reporting for specific materials, systems and methods

We require information from authors about some types of materials, experimental systems and methods used in many studies. Here, indicate whether each material, system or method listed is relevant to your study. If you are not sure if a list item applies to your research, read the appropriate section before selecting a response.

Materials & experimental systems

n/a	Involved in the study	
☒	☐	Antibodies
☐	☒	Eukaryotic cell lines
☒	☐	Palaeontology and archaeology
☒	☐	Animals and other organisms
☒	☐	Clinical data
☒	☐	Dual use research of concern

Methods

n/a	Involved in the study	
☒	☐	ChIP-seq
☒	☐	Flow cytometry
☒	☐	MRI-based neuroimaging

Eukaryotic cell lines

Policy information about cell lines and Sex and Gender in Research

Cell line source(s)	Sf9 cells were used for recombinant expression of the human Ku70/ku80 heterodimer. Commercial Source: Gibco(TM) (ThermoiFisher Scientific) (Reference number: 11496015)
Authentication	The cell line was not authenticated.
Mycoplasma contamination	The cell lines were not tested for mycoplasma contamination.
Commonly misidentified lines (See ICLAC register)	No commonly misidentified cell lines were used in the study.
