# Supplementary material for: Structural basis for the inactivation of cytosolic DNA sensing by the vaccinia virus
Source: Nat Commun. 2022 Nov 18;13:7062. doi: 10.1038/s41467-022-34843-z (PMC9674614; doi:10.1038/s41467-022-34843-z)
Supplement: Supplementary file 8 — Source Data [file 41467_2022_34843_MOESM8_ESM.zip › Source_Data_for_Figure2b.pdf]

Source Data for SDS-PAGE shown in Figure 2b

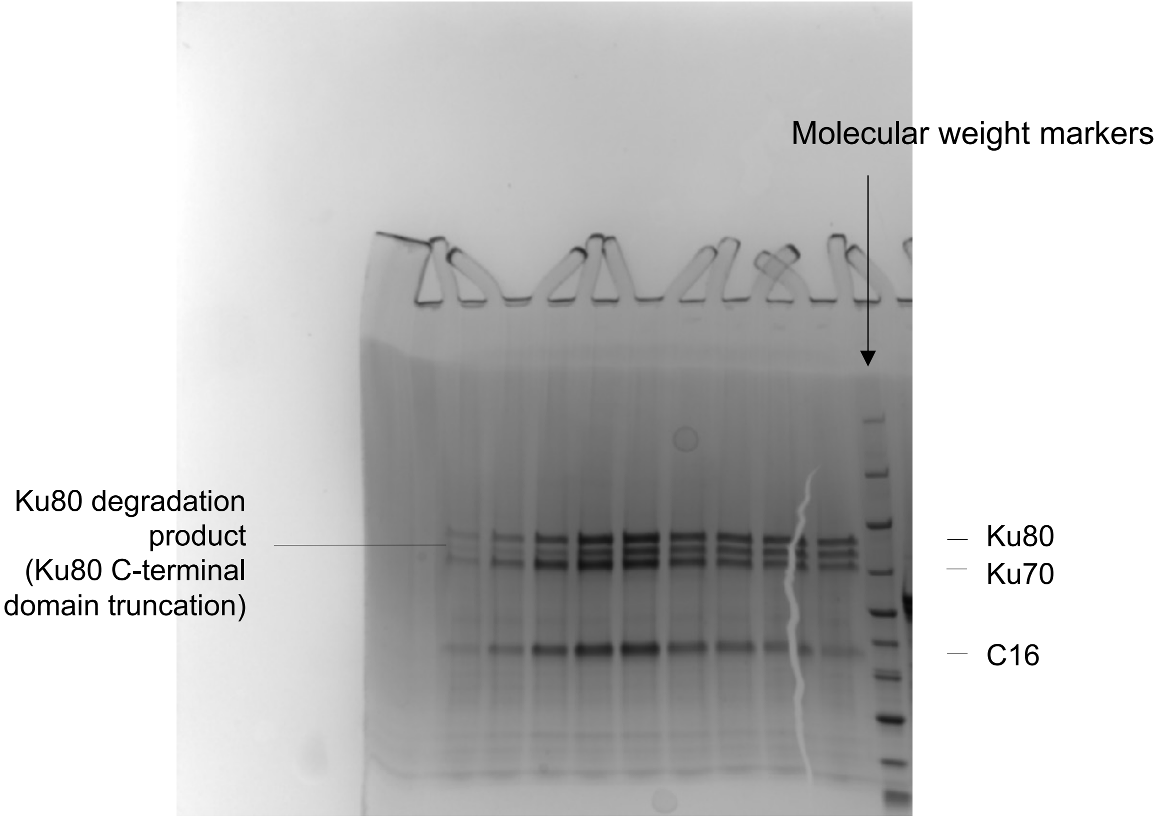

Fractions from Size Exclusion Chromatography in Figure 2a –
Purification of the
C16 – Ku complex

Fractions of the size exclusion chromatography for purification of the C16 – Ku complex. Ku80 has a tendency to proteolysis, losing the C-terminal domain that does not form part of the core of the Ku70/Ku80 dimer, as described in previous works by several authors.

For cryoEM analysis, fractions with a minor presence of the Ku80 truncation were selected.
